# Curriculum Innovation: A Leadership Training Curriculum for Neurology Residents

**DOI:** 10.1212/NE9.0000000000200353

**Published:** 2026-09-23

**Authors:** Deborah Young Bradshaw, Shahram Izadyar, Dongliang Wang, Corey McGraw, Julius Gene Silva Latorre, Anuradha Duleep, Lauren Germain, Jenny A. Meyer, Laura E. Simionescu, Michael Vertino

**Affiliations:** 1 Department of Neurology, SUNY Upstate Medical University, Syracuse, NY; and; 2 Department of Public Health and Preventive Medicine, SUNY Upstate Medical University, Syracuse, NY.

## Abstract

**Background and Objectives:**

Physicians assume leadership roles in health care, starting during their residency. Despite the need for skilled physician leaders, medical students and residents seldom receive training in how to lead. We aimed to create a leadership training curriculum to equip our neurology residents with knowledge and skills to lead their health care teams during residency and beyond. The learning objectives, in summary, were to improve self-awareness, demonstrate effective communication, improve conflict management, build personal sustainability, develop feedback and mentoring skills, engage in health care systems improvement, and strengthen leadership identity.

**Methods:**

We created a 3-year leadership training program for neurology residents at State University of New York (SUNY) Upstate Medical University. Interested faculty members holding leadership positions in the Neurology Department formed the Leadership Academy faculty and developed the curriculum. Sessions included didactic presentations followed by active learning exercises, including small group discussion, writing exercises, and role-playing, followed by group reflection. We used 3 assessment tools: (1) individual session evaluation for quality control and program improvement; (2) a leadership self-assessment for physicians developed by the National Health Service (NHS), modified for relevance to residents and deployed annually to all residents; and (3) resident self-assessments conducted at the end of each academic year end-of-year assessment to collect resident's perceptions of their knowledge and leadership behaviors before and after exposure to the curriculum.

**Results:**

Grouping all residents together, no significant differences from year 1 to year 3 were observed on the NHS self-assessment. However, 7 residents who completed the NHS self-assessment in 3 consecutive years demonstrated statistically significant improvements in their comfort level managing conflict (*p* = 0.013) and in engaging with others to determine the direction of the program, department, and university (*p* < 0.001). Numerous statistically significant improvements in leadership knowledge and self-assessed leadership behaviors were observed in the end-of-year assessments.

**Discussion:**

We successfully implemented a leadership training curriculum for neurology residents.

## Introduction

Physicians become de facto leaders the day they graduate from medical school. During the few weeks between the end of medical school and the beginning of their internship, they transition from a peripheral role on the health care team to a central one. In academic medical centers, interns serve as the front-line providers of patient care and are often the only physicians available after hours. Patients, families, nurses, medical students, and allied health professionals turn to them for expertise and direction. As they progress through residency, they take on a larger leadership role, supervising the work of junior residents. Some hold formal leadership positions within residency, such as chief resident. After graduation, those entering both private practice and academic medicine frequently assume leadership roles within their practice, section, or educational program. Eventually, many physicians take on leadership roles within their specialty at the regional or national levels. Physicians also play leadership roles in the broader health care industry, where they are generally regarded as credible leaders. Examples include designing health care protocols, allocating human resources, and ensuring compliance with or advocating for modifications of regulations. Some go on to lead hospital systems or government agencies involved with public health or health care regulation. Physician leaders are instrumental in establishing the values, mission, and vision of health care organizations, and they contribute to fostering cultures of accountability and excellence. Leadership competencies expected of physician leaders include knowledge of the industry and technical skills, emotional intelligence, vision, organizational orientation, collegial respect, trust, fairness, two-way communication skills, regular feedback, and problem-solving skills.

Despite the many leadership responsibilities inherent in the work of physicians, leadership training is not a core curricular element in either undergraduate or postgraduate medical education.^1^ Numerous authors have drawn attention to this deficit and called for formal leadership training programs.^2,3^ An editorial in Journal of Graduate Medical Education stated “an individual resident's exposure to and opportunity for personal growth in leadership skills varies from extensive to none.”^4^ The Accreditation Council for Graduate Medical Education (ACGME) Milestones incorporate numerous leadership skills at the higher levels, but programs are left to themselves to create curricula and assessments to help residents develop them. Indeed, there is evidence suggesting that residents are not prepared for leadership roles upon graduation,^5^ and many physician leaders become “accidental leaders” rather than deliberately choosing to lead.^6^

Physicians also require good leaders. A strong inverse relationship has been demonstrated between good quality leadership for physicians and the level of burnout they experience. High burnout levels negatively affect quality of care.^7^ Finally, there is ample evidence that leadership development for physicians affects the quality of patient care directly.^8,9^ For these reasons and others, the health care industry needs a pipeline of physicians who identify as leaders, who are practiced and confident in their leadership skills, and who are committed to lifelong development as leaders.

The Leadership Identity Development Model (LID) is one of the most frequently applied frameworks in higher education for understanding how individuals come to see themselves as leaders. The model outlines 6 stages of development through which individuals progress in a cyclical and nonlinear fashion, shaped by their experiences, reflection, and ongoing learning.^10^ The latter stages of the model emphasize using leadership to positively influence one's environment and align personal identity with broader organizational or community goals. These stages are particularly relevant for residents, whose leadership responsibilities increasingly involve shaping team dynamics, advancing patient care, and contributing to the culture of their training programs.

Intentional leadership development programming facilitates movement between LID stages, helping trainees build the self-awareness, skills, and confidence necessary to progress. A recent review highlights the need for integrating leadership training to optimize leadership development programs and ensure they are responsive to the complex realities of health care practice.^11^

In summary, there is growing interest in incorporating formal leadership development into medical education.^12^

### Objectives

To develop and deliver a leadership training curriculum to neurology residents and enhance their confidence and skill in leading health care teams. The overall program goal and individual learner objectives of the curriculum are presented in Table 1.

**Table 1:** Gilbert S. Ross Leadership Academy Learning Goals and Objectives

**Goal**
To accelerate the acquisition of leadership skills in neurology residents
**Objectives**
(1) Improve self-awareness and reflective practice (2) Demonstrate effective communication and team collaboration (3) Recognize and manage conflict in the workplace (4) Build personal sustainability, ethics, and professional purpose (5) Develop feedback, teaching, and mentoring skills (6) Engage in health care systems awareness and quality improvement (7) Strengthen leadership identity and strategic thinking

## Methods

Faculty members holding departmental leadership roles, including program directors, clinic directors, quality officers, and section heads, were invited to convene as the leadership curriculum committee. Those with an interest formed the core leadership curriculum faculty. We first defined the role of the curriculum committee and then created a vision and mission for the program, which we titled, “The Leadership Academy (LA).” A literature search was conducted to identify leadership training methods and best practices, and residents and LA faculty were polled for topics of interest.

A tentative 3-year schedule was sketched. Faculty members selected topics to present, based on their own interests and background. Session topics for each year, their related curriculum goals and objectives, and mapping to the ACGME milestones for each year are presented in Tables 2–4. Formatting of the mentioned tables and mapping to the milestones, goals and objectives were completed with the assistance of OpenAI's GPT-5.3 language model, and the authors independently validated the results.

**Table 2:** Gilbert S. Ross Leadership Academy at Upstate Medical University, First-Year Curriculum, July 2019–June 2020

Topic	G and O^a^	ACGME competencies^b^	Milestone focus	Potential outcomes
Special grand rounds: guest speaker, Dr. Terrence Cascino (former president of American Academy of Neurology)	7	PROF, SBP	Professional identity	Identifies importance of leadership development for neurologists
What makes a leader? Role of emotional intelligence	1, 7	PROF, SBP	Identity; reflection	Identifies strengths/limits; begins leadership identity
NHS leadership assessment	1	PBLI, SBP	Self-assessment	Completes assessment; drafts development plan
Preparing for fellowship	4, 7	PROF	Career development	Aligns career goals with values
Organization: getting things done	2, 6	SBP, ICS	Team function; efficiency	Demonstrates prioritization and workflow awareness
Teaching and mentoring	2	ICS	Teaching	Participates in teaching; receives feedback
Emotional intelligence: the neuroscience	1, 4	PROF	Self-regulation	Describes emotional awareness in clinical context
Effective listening	2, 3	ICS	Communication	Demonstrates active listening
Leadership styles	1, 7	PROF	Adaptability	Identifies leadership style
Providing effective feedback	5	ICS, PBLI	Feedback	Practices structured feedback
Burnout and wellness	4	PROF	Well-being	Identifies stressors and coping strategies
NHS review + annual evaluation	All	PBLI, PROF	Reflection	Updates development plan; reflects on growth

Abbreviations: ACGME = Accreditation Council for Graduate Medical Education; NHS = National Health Service.

a Goals and objectives as presented in Table 1.

b PROF: Professionalism; ICS: Interpersonal and Communication Skills; PBLI: Practice-Based Learning and Improvement; SBP: Systems-Based Practice.

**Table 3:** Gilbert S. Ross Leadership Academy at Upstate Medical University, Second-Year Curriculum, July 2020–June 2021

Topic	G and O^a^	ACGME competencies^b^	Milestone focus	Potential outcomes
NHS review + annual evaluation	1, 7	PBLI, PROF	Reflection; identity formation	Updates development plan; reflects on leadership growth
Racism in American medicine	4, 6	PROF, SBP	Professional responsibility; systems awareness	Describes impact of structural inequities on care and teams
Mentoring	2, 5	ICS	Teaching and mentorship	Demonstrates mentoring behaviors with junior learners
Transformative leadership: guest speaker, Dr. Jennifer Bickel	6, 7	PROF, SBP	Leadership identity	Articulates evolving leadership approach
Pick the chair's brain	7, 6	SBP, PROF	Systems leadership	Identifies institutional leadership challenges
Building emotional intelligence	1, 4	PROF	Self-regulation	Applies emotional awareness in team settings
Difficult conversations	2, 3	ICS, PROF	Communication; conflict management	Demonstrates structured approach to difficult discussions
Conflict resolution (multisession series): Guest speaker	3	ICS, PROF	Conflict management	Applies conflict resolution strategies
Top teams	2, 6	SBP, ICS	Team function	Describes characteristics of high-performing teams

Abbreviation: ACGME = Accreditation Council for Graduate Medical Education.

a Goals and objectives as presented in Table 1.

b PROF: Professionalism; ICS: Interpersonal and Communication Skills; PBLI: Practice-Based Learning and Improvement; SBP: Systems-Based Practice.

**Table 4:** Gilbert S. Ross Leadership Academy at Upstate Medical University, Third-Year Curriculum, July 2021–June 2022

Topic	G and O^a^	ACGME competencies^b^	Milestone focus	Potential outcomes
NHS review + annual evaluation	1, 7	PBLI, PROF	Reflection; integration	Demonstrates longitudinal growth; refines leadership philosophy
Teams 2: Five dysfunctions of a team	2, 6	ICS, SBP	Team leadership	Analyzes and addresses team dysfunction
Servant leadership	4, 7	PROF	Leadership identity	Articulates values-based leadership approach
Conflict resolution: real-life vignettes (parts 1 and 2): including guest speaker	3	ICS, PROF	Advanced conflict management	Manages complex team conflict scenarios
Diversity and inclusion	4, 6	PROF, SBP	Professional responsibility; systems awareness	Applies inclusive leadership principles
Managing your energy	4	PROF	Sustainability	Demonstrates strategies for sustained performance
Positivity science	4	PROF	Well-being	Applies cognitive/emotional strategies
Leading effective meetings	2, 6	ICS, SBP	Team leadership	Leads structured, goal-oriented meetings
Storytelling: giving a great lecture	2, 5	ICS	Communication; teaching	Delivers organized, engaging teaching
Work/life balance: guest speaker, Dr. Carlayne Jackson (former president of American Academy of Neurology)	4	PROF	Professional sustainability	Integrates long-term career sustainability strategies

Abbreviations: ACGME = Accreditation Council for Graduate Medical Education; NHS = National Health Service.

a Goals and objectives as presented in Table 1.

b PROF: Professionalism; ICS: Interpersonal and Communication Skills; PBLI: Practice-Based Learning and Improvement; SBP: Systems-Based Practice.

Most (84.4%) sessions were delivered by the LA faculty, 1 session (3.1%) by a non-neurologist physician outside the neurology department, and 4 sessions (12.5%) by guest speakers from outside the institution, 3 of whom were neurologists.

We substituted 1-hour monthly sessions for the regular resident training curriculum, which includes approximately 20 didactic hours/mo in addition to departmental Grand Rounds. The course content spanned 3 years. Therefore, each resident was offered 36 contact hours of leadership training over the 3-year residency program. Residents at the postgraduate year (PGY)-2 through PGY-4 levels participated in the same sessions. In addition, we held 1-hour preparatory sessions several weeks before each live session for the faculty member to present and receive feedback on their presentations from other LA faculty. Presentations were a mix of didactic and interactive elements and included online self-assessments, role plays, and writing exercises. Samples of the interactive elements and other activities are presented in Table 5.

**Table 5:** Samples of Session Activities

Topic	Lecture	Activity
Time management	• Principles of time management • Chief resident presents practical tips for organizing workday in hospital • Managing email	• Residents complete worksheet of their activities for the past 24 h • Residents create task list including priority and assign tasks to each quadrant of Eisenhower Matrix
Giving feedback	• Principles of ADAPT^a^ feedback model	• Two faculty members do a live impromptu feedback with written scenario using ADAPT method • Discussion • Residents break into groups of 3 and use scenarios to practice feedback to student, peer, and/or supervisor
Conflict management (2 sessions)	• Costs of conflict in medical settings (errors, burnout) • Phases of conflict and interventions to abort it • Self-calming techniques; prep for discussion after conflict • Suggested language and body language	• Writing exercise: past conflict, causes, resolution, lasting impacts • Discussion • Case discussion with analysis of conflict phases
	• Quick review of conflict management principles	• Real conflict scenarios submitted by residents • Role plays in groups of 3 managing the conflicts
Building your emotional intelligence (EI)	• Neurobiology of EI • Naming emotions • Methods for building EI	• Residents take free online quiz to measure their EI in session • Find accountability partner and set EI development goals
Annual leadership self-assessment	• None	• Residents complete NHS survey before session and bring printouts • Residents answer worksheet before session • Break into PGY classes and discuss their progress and worksheet questions • Each class creates a SMART^b^ goal for improvement of leadership skills over the next year record • In 1 year, they check in on their progress and report to group

Abbreviation: NHS = National Health Service.

a ADAPT: Ask, Discuss, Ask again, Plan Together.^13^

b Specific, measurable, achievable, relevant, and time bound.

Evaluation methods were 3-fold. First, a leadership self-assessment for physicians developed by the National Health Service (NHS)^14^ was modified for relevance to residents. The modified NHS Leadership Self-Assessment includes 6 domains: demonstrating personal qualities (i.e., I remain calm under pressure), working with others (i.e., I identify opportunities to collaborate with others to improve patient care), managing services (i.e., I take action when resources are not being used efficiently and effectively), improving services (i.e., I encourage debate about new ideas with a wide range of people), setting direction (i.e., I use data and information to suggest improvements to services), and creating the vision (i.e., I actively engage with others to determine the direction of the program, department, and university). We administered the survey to all Neurology residents at baseline and after each year of training. We term this the longitudinal survey data. All surveys to participants were anonymous.

Following the first year of administration, the faculty determined that the survey was excessively long, and we held a special meeting to edit the NHS self-assessment tool. We eliminated 11 questions, primarily those pertaining to high-level health care management. In some cases, we simplified the wording of questions retained in subsequent surveys.

Individual session evaluations after each presentation were our second assessment method. They were administered on the day of the educational sessions and asked the residents to evaluate the quality of the session, including 2–3 questions to determine whether the session achieved its specific learning goals. These were used to assess session quality and to modify content during subsequent cycles.

Finally, assessments were conducted at the end of each academic year end-of-year assessment (EYA). The final session of each year consisted of a review of the year's curriculum, highlighting key learning points from each session. End-of-year assessments were deployed shortly after the year in review. They composed of several questions drawn from the NHS leadership survey, several original questions pertaining to leadership development, and several questions pertaining specifically to that year's curricular content. The questions asked the residents to assess their understanding of leadership concepts or leader behavior before and after the sessions. Between years 1 and 2 the EYAs were changed from a 4 to a 5-point Likert scale.

Categorical variables and Likert scale variables were presented in terms of total number and percentage. Mean scores of the Likert scale variables were also plotted to examine the trend over time. Differences between the 3 years were assessed using 1-way analysis of variance. Differences between the presessions and postsessions of each year were assessed using paired *t* test. No correction for multiple comparisons was performed. Statistical analyses were conducted using R version 4.1.3, and a *p* value <0.05 was considered statistically significant.

The Leadership Identity Development (LID) Model provides a useful framework for understanding the design and evaluation of the Leadership Academy. The program structure intentionally recognized that residents are at different stages of leadership development and may cycle through stages multiple times as they encounter new roles and challenges. By combining multiple methods of development facilitation from didactic instruction to interactive role plays, reflective writing, and self-assessments, the curriculum created opportunities for residents to move to the more advanced stages of growth, impact, and synthesis. The longitudinal evaluation strategy further aligns with LID's recognition that leadership identity develops progressively through experience, feedback, and reflection.

### Standard Protocol Approvals, Registrations, and Patient Consents

The Upstate Medical University Institutional Review Board determined this protocol to be exempt under federal regulation 45 code of feneral regulations 46.104(d) (4).

### Data Availability

Anonymized data not published within this article will be made available by request from any qualified investigator.

## Results

Attendance of the residents was monitored during the 3-year curriculum. Thirty-two (100%), 27 (79%), and 19 (29%) residents completed the annual NHS self-assessment in 2019, 2020, and 2021, respectively. The response rates for completing year-end and presession and postsession surveys in the same years were 16 (52%), 23 (68%), and 10 (29%). Grouping all residents together each survey year, no significant differences from year 1 to year 3 were observed in the longitudinal survey data. Additional data are listed in eTable 1.

Seven residents completed the NHS survey in 3 consecutive years. Over the 3-year period, responses from the 7 residents demonstrated a significant difference on 2 questions, “I am comfortable managing conflicts of interest or differences of opinion” (*p* = 0.013) and “I actively engage with others to determine the direction of the program, department, and university” (*p* < 0.001). In year 1 of the curriculum, 14.3% endorsed the statement, “I am comfortable managing conflicts of interests or differences of opinion” “a lot of the time,” while at the end of year 3, 71.4% of the same respondents endorsed this statement. Similarly, in year 1, 14.3% endorsed the statement I actively engage with others to determine the direction of the program, department, and university a lot of the time, while at the end of year 3, 84% endorsed the same statement. The statistically significant results are presented in Table 6 and the Figure, and complete survey results are listed in eTable 2. No other significant differences were observed.

**Table 6:** Longitudinal Survey Data in Participants With 3-Year Data

Question	3-Point Likert Scale	Year, weighted average (%)			*p* Value
		2019-2020 (N = 7)	2020-2021 (N = 7)	2021-2022 (N = 7)	
Working with others					
I identify opportunities to collaborate with others to improve patient care	A lot of the time	57.1	57.1	71.4	0.385
	Most of the time	28.6	42.9	28.6	
	Very little/none of the time	14.3	0	0	
I communicate clearly and effectively with others	A lot of the time	71.4	42.9	85.7	0.593
	Most of the time	28.6	57.1	14.3	
	Very little/none of the time	0	0	0	
I listen to and take into account the needs and feelings of others	A lot of the time	100	66.7	71.4	0.201
	Most of the time	0	33.3	28.6	
	Very little/none of the time	0	0	0	
I actively seek contributions and views from others	A lot of the time	57.1	42.9	57.1	1
	Most of the time	28.6	57.1	28.6	
	Very little/none of the time	14.3	0	14.3	
I am comfortable managing conflicts of interests or differences of opinion	A lot of the time	14.3	71.4	71.4	**0.013**
	Most of the time	57.1	28.6	28.6	
	Very little/none of the time	28.6	0	0	
I put myself forward to lead teams? I acknowledge the contributions of teammates	A lot of the time	42.9	42.9	42.9	0.545
	Most of the time	14.3	28.6	42.9	
	Very little/none of the time	42.9	28.6	14.3	
Creating the vision					
I actively engage with others to determine the direction of the program, department, and university	A lot of the time	14.3	28.6	85.7	**<0.001**
	Most of the time	42.9	71.4	14.3	
	Very little/none of the time	42.9	0	0	
I challenge behaviors, symbols and rituals which are not consistent with Upstate's vision	A lot of the time	42.9	42.9	57.1	0.148
	Most of the time	14.3	57.1	42.9	
	Very little/none of the time	42.9	0	0	

Only domains with at least 1 statistically significant item are shown (bold). Complete data are presented in eTable 2.

**Figure:**
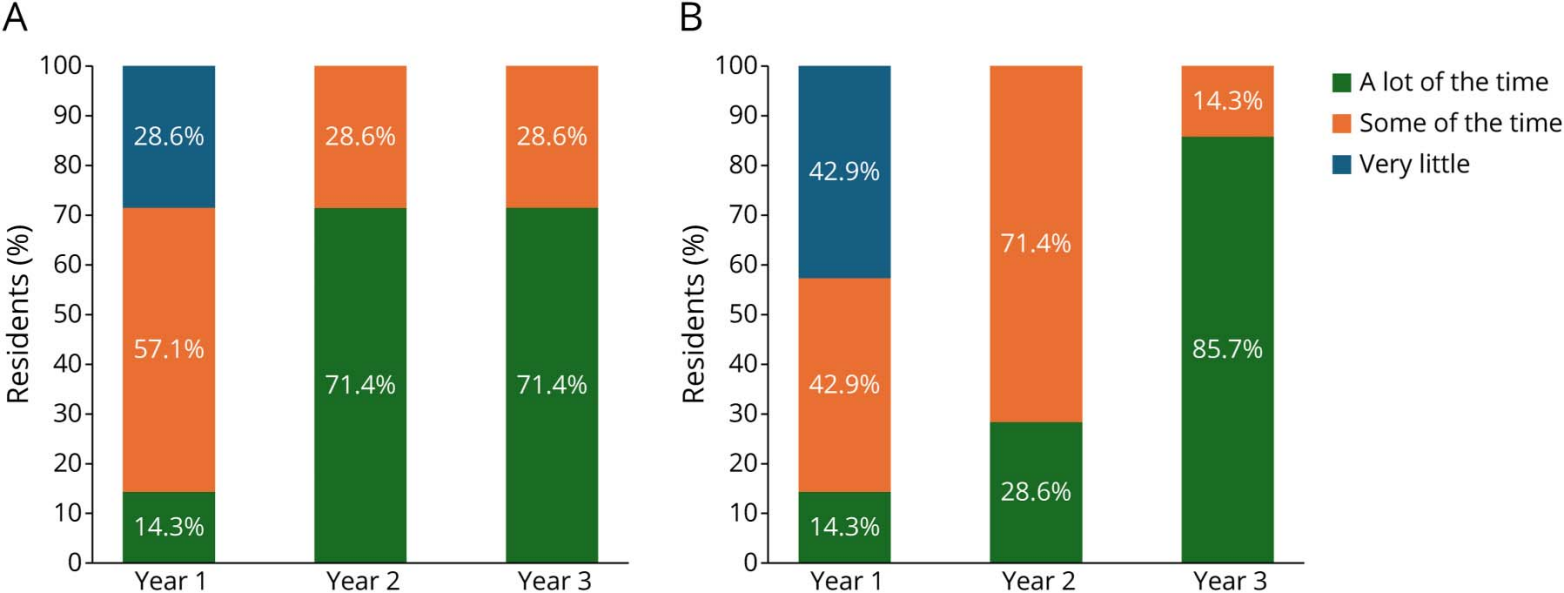
Statistically Significant Items in Longitudinal Survey Data in Participants With 3-Year Data (A) I am comfortable managing conflicts of interest or differences of opinion (*p* = 0.013). (B) I work with others to determine the direction of the program, department, and university (*p* < 0.001).

By contrast, numerous significant differences were detected in the end-of-year (EYA) survey data. The results are grouped using the NHS domains and are further divided into (1) NHS questions, (2) original questions written by the LA faculty and (3) questions pertaining directly to session content (each session was given once over 3 years). Complete EYA survey data are presented in eTables 3 and 4. The NHS domains include demonstrating personal qualities, working with others, managing services, improving services, setting direction, and creating the vision and advancing aims. Each domain has several subcategories. We grouped the 3 types of questions this way to determine whether growth was more evident in some domains than others. Only 3 EYA questions failed to show significant change: First, “I apply leadership skills in my daily work” in year 1, although significant differences were evident on this question in the subsequent 2 years. Second, “I put myself forward for leadership positions” did not reach significance in year 3, although it did in years 1 and 2. Finally, “I put forward ideas to improve patient care” was significant in years 2 and 3 but not in year 1.

The highest levels of significance (*p* < 0.0001) occurred on the original questions—those written by the LA faculty to assess resident engagement in leadership development. Anecdotally, one of the key changes we observed in the residents was their awareness of themselves as leaders. That is, although they were functioning in leadership roles, they did not explicitly identify as leaders until this curriculum was offered. The mapping of learning objectives to improvements in self-assessment is presented in eTable 5.

### Discussion

Physicians assume multiple leadership roles, yet they receive little formal training in leadership during their undergraduate or graduate medical education. Two systematic reviews summarized the literature on leadership training in graduate medical education. In 1 review, Frich et al.^15^ identified 45 articles published between 1989 and 2014 that described the outcomes of programs designed to expose physicians to leadership concepts. Fifty-eight percent of these programs targeted residents or fellows, but only 3 were designed for residents who already held or were expected to hold a leadership role such as chief residents. More than 70% of the programs were structured as extended courses delivered over time, with only 6 (13.3%) having curricula longer than 1 year. Fifty-six percent of the articles reported learner satisfaction, 80% indicated a subjective increase in knowledge, 16% showed an objective increase in knowledge, and 26% noted behavior change.

In a separate systematic review, Sadowski et al. identified 52 articles that described the outcomes of programs teaching leadership in graduate medical education^2^ (18 of these articles were also included in the previously mentioned systematic review). They found that 8 programs (15%) were designed solely for chief residents. Twelve (23%) of the studies were single sessions, 19 (37%) were serial lessons delivered over a period, and 21 (40%) were longitudinal curricula. Reported outcomes were similar to those of the previous systematic review, with 54% of articles reporting learner satisfaction, 50% reporting a subjective increase in knowledge, 13% noting an objective increase in knowledge, and 15% indicating behavior change. These systematic reviews indicate that while many departments and programs have attempted leadership development, few have focused on residents other than those defined as resident leaders, such as Chief Residents. The programs vary in duration and intensity, and outcomes are mostly limited to learner satisfaction.

The American Academy of Neurology has spearheaded leadership training for neurologists over the past decade, developing numerous programs, particularly aimed at advancing the leadership skills of underrepresented groups. However, the American Academy of Neurology programs focus on neurology physicians already in practice. A deficit still exists in leadership development for trainees. To address this gap in leadership training for neurology residents, we created a longitudinal leadership training program for them. Resident self-assessments demonstrated significant increases in both knowledge and self-reported behaviors across multiple leadership domains. In addition, the sessions were well-attended and received positive evaluations from residents.

The culture of residency creates several significant barriers to yet another training requirement. First, the extraordinary physical, cognitive, and emotional demands of residency pose a challenge, leaving many residents feeling unable to take on additional work, such as leadership training. In addition, such programs may be perceived as “soft” or burdensome in the context of heavy clinical workloads. Furthermore, the lengthy and hierarchical training of physicians, their extensive individual-based performance evaluations during training, and their “deficit-based” way of thinking in approaching differential diagnosis may hinder the development of collaborative instincts. Finally, acknowledging the numerous competencies that physicians must master, the ACGME has gradually increased the training requirements for residency programs to encompass aspects such as evidence-based medicine, cost-consciousness, and ethics. Thus, the culture of residency presents numerous barriers to yet another training requirement.

Leadership training for physicians can be understood as the integration and intentional development of competencies already embedded within graduate medical education. Many skills essential to effective physician leadership—such as interpersonal and communication skills, systems-based practice, and quality improvement—are core ACGME Milestones. For example, although interpersonal and communication skills are fundamental to clinical care, they are equally critical to leading teams. Similarly, quality improvement initiatives, a required component of residency training, demand sophisticated leadership to be successful. Despite this overlap, residency programs often struggle to teach these competencies in a practical, applied manner. A longitudinal leadership curriculum does not duplicate existing training; rather, it provides a focused framework that integrates, contextualizes, and advances these competencies as observable leadership behaviors. The curriculum readily maps to the ACGME Milestones, as presented in Tables 2–4.

This study has several limitations. The attrition in survey completion is noteworthy. The percentage of residents completing both the NHS self-assessment and the end-of-year assessment decreased significantly over the first 3-year cycle. In year 1, 100% of residents completed the NHS self-assessment, whereas only 29% completed it by the end of year 3. Similarly, 52% of residents completed the year-end survey in year 1, while only 29% completed it by the end of year 3. The decline in survey completion can be attributed to fewer reminders from the program director as the program progressed.

Other study limitations include the experience of a single institution and the lack of a control group. In addition, the survey instruments were modified between year 1 and year 2 of the curriculum for simplification. Thus, comparing NHS survey results between years 1 and 2 may not be accurate. Finally, and possibly most importantly, outcome measures relied on resident self-assessment rather than evaluations by faculty, peers, or other team members. Future evaluations of our program's success will include more objective outcomes that can be tied to program goals.

Residents accrue leadership skills naturally as they progress through residency, even in the absence of formal training. To establish that their growth as leaders can be attributed to the Leadership Academy program would require comparison with a group of residents in training who accrued leadership skills naturally and as a function of their progression through residency.

Opportunities for curriculum development include incorporating our Neurology residents into session development and delivery to a greater extent. We anticipate that this would increase resident engagement in the content and generate additional topics to cover. In addition, we plan to add curricular content that addresses areas of weaker growth on the NHS self-assessment tool, such as using patient feedback and collaborating with colleagues to drive system change.

Over the past 6 years, we have noticed that resident applicants are very interested in our Leadership Academy; in fact, some residents arrive for their PGY-1 year reporting that they selected our program because of our Leadership Academy. In the future, we may leverage this advantage by offering a residency track and/or fellowship in Leadership in Neurology. In addition, we see an opportunity to lead other programs in developing similar curricula. Several residency programs in Neurology and other specialties have expressed interest in implementing our model in their own programs.

Anecdotally, our Chair and the Leadership Academy faculty have observed that preparing and presenting this material has had a positive impact on the leadership behaviors of our faculty. We have begun adapting the LA sessions for use as faculty development sessions.

Opportunities to improve evaluation of program effectiveness include partnering with other residency programs to determine whether our curriculum accelerates leadership development beyond that naturally achieved during residency. Measures of resident leadership behavior observed by peers, students, and faculty before and after leadership training would be a convincing metric of success. Finally, capturing such outcomes as the number of leadership roles attained and success in leadership after residency would be the gold standard of success we hope to achieve.

We successfully integrated formal leadership training into our neurology residency program. Resident self-assessments demonstrated improvements over 3 years in multiple domains. Moreover, our residents have embraced it and attest to its value to their daily practice. It attracts applicants to both our residency and our faculty. Finally, as the faculty members delivering the content and participating with our residents in the live sessions, we experience benefits to our own leadership development and wellness.

## Glossary

ACGME: Accreditation Council for Graduate Medical Education
EYA: end-of-year assessment
LA: Leadership Academy
LID: Leadership Identity Development Model
NHS: National Health Service
